# Characterization of *Lactobacillus salivarius* alanine racemase: short-chain carboxylate-activation and the role of A131

**DOI:** 10.1186/s40064-015-1335-6

**Published:** 2015-10-24

**Authors:** Jyumpei Kobayashi, Jotaro Yukimoto, Yasuhiro Shimizu, Taketo Ohmori, Hirokazu Suzuki, Katsumi Doi, Toshihisa Ohshima

**Affiliations:** Microbial Genetics Division, Institute of Genetic Resources, Faculty of Agriculture, Kyushu University, Fukuoka, 812-8581 Japan; Applied Molecular Microbiology and Biomass Chemistry Bioscience and Biotechnology, Faculty of Agriculture, Kyushu University, Fukuoka, 812-8581 Japan; Department of Biomedical Engineering, Faculty of Engineering, Osaka Institute of Technology, 5-16-1 Omiya, Asahi-ku, Osaka, 535-8585 Japan; Functional Genomics of Extremophiles, Faculty of Agriculture, Graduate School, Kyushu University, Fukuoka, 812-8581 Japan; Department of Biomedical Engineering, Faculty of Engineering, Osaka Institute of Technology, 5-16-1 Ohmiya, Asahi-ku, Osaka, 535-8585 Japan

**Keywords:** d-amino acid, Alanine racemase, *Lactobacillus salivarius*, Lactic acid bacteria, Short-chain carboxylate

## Abstract

Many strains of lactic acid bacteria produce high concentrations of d-amino acids. Among them, *Lactobacillus salivarius* UCC 118 produces d-alanine at a relative concentration much greater than 50 % of the total d, l-alanine (100d/d, l-alanine). We characterized the *L. salivarius* alanine racemase (ALR) likely responsible for this d-alanine production and found that the enzyme was activated by carboxylates, which is an unique characteristic among ALRs. In addition, alignment of the amino acid sequences of several ALRs revealed that A131 of *L. salivarius* ALR is likely involved in the activation. To confirm that finding, an *L. salivarius* ALR variant with an A131K (ALR^A131K^) substitution was prepared, and its properties were compared with those of ALR. The activity of ALR^A131K^ was about three times greater than that of ALR. In addition, whereas *L. salivarius* ALR was strongly activated by low concentrations (e.g., 1 mM) of short chain carboxylates, and was inhibited at higher concentrations (e.g., 10 mM), ALR^A131K^ was clearly inhibited at all carboxylate concentrations tested (1–40 mM). Acetate also increased the stability of ALR such that maximum activity was observed at 35 °C and pH 8.0 without acetate, but at 50 °C in the presence of 1 mM acetate. On the other hand, maximum ALR^A131K^ activity was observed at 45 °C and around pH 9.0 with or without acetate. It thus appears that A131 mediates the activation and stabilization of *L. salivarius* ALR by short chain carboxylates.

## Background

With the exception for glycine, which contains no asymmetric carbon, all proteogenic amino acids exist as l-α-form molecules. Indeed, up to around 1980, d-amino acids as enantiomers of corresponding l-amino acids were generally considered to have no important role in living organisms, and so attracted little attention, though it was acknowledged that some d-amino acids were among the main constituents of bacterial cell walls (Heijenoort 2001). However, advances in the analytical techniques used to study amino acid enantiomers have shown that, in fact, d-amino acids are widely distributed among a variety of organisms, including plants, fish and mammals (Kullman et al. 1999; Erbe and Brückner. 2000; Pätzold et al. 2005). In addition, it was recently reported that d-amino acids exert inhibitory effects on biofilm formation (Kolodkin-Gal et al. 2010; Hochbaum et al. 2011). Such evidence of the physiological importance of d-amino acids has prompted investigation into their metabolism and the enzymes involved.

d-Alanine is well known to be a component of bacterial cell walls and to be synthesized from l-alanine by alanine racemase (ALR). Our group recently found that many lactic acid bacteria secrete d-alanine into their medium (Mutaguchi et al. 2013) and the relative percentage (100d/(d + l)) of d-alanine per total d, l-alanine in many lactic acid bacterial cells is more than 50 %; the value for *Lactobacillus salivarius* is 89.6 %, for example. ALR is thought to be pivotal for d-alanine production in lactic acid bacteria, but this racemase generally catalyzes the conversion of l-alanine to a racemic mixture, and the d-alanine percentage does not normally exceed 50 %. Thus it may be postulated that an unknown mechanism accounts for the excess d-alanine production seen in *L. salivarius*.

We previously identified a d-amino acid aminotransferase (d-AAT) in *L. salivarius* that exhibited characteristics different from those of known d-AATs from strains of *Bacillus* and *Geobacillus* species (Kobayashi et al. 2013). In that context, we were interested in the inherent characteristics of ALR of *L. salivarius* and identified an ALR homolog gene in an *L. salivarius* data base. In the present study, we expressed this *L. salivarius* ALR gene in *Escherichia coli* and, interestingly, found that the purified ALR protein was strongly activated in the presence of carboxylates such as acetate and propionate. By contrast, ALRs from *Geobacillus* and *Bacillus* species are reportedly inhibited by acetate and propionate (Morollo et al. 1999; Kanodia et al. 2009). In addition, ALRs from *L. fermentum* and *Pseudomonas putida* are known to be stabilized by acetate, although the mechanism for this effect is not yet known (Johnston and Diven. 1969; Rosso et al. 1969). We therefore compared the primary sequences of ALRs from *L. salivarius*, *L. fermentum* and *P. putida* with those of various *Bacillus* and *Geobacillus* species. Nearly all important residues for the execution of catalytic activity are well conserved, except for a single residue: A131 in *L. salivarius*, *L. fermentum* and *P. putida* ALRs, and K129 in *Bacillus* and *Geobacillus* ALRs. K129 is thought to stabilize R136 binding to the carboxylate group through carbamate formation (Morollo et al. 1999). Here we examined the role of A131 in the regulation of *L. salivarius* ALR by the carboxylates by preparing an *L. salivarius* ALR variant (A131K; ALR^A131K^) and comparing its properties with those of the parental wild-type ALR.

## Methods

### Sequence analysis of ALR

The primary amino acid sequence of *L. salivarius* ALR was analyzed and compared with those of previously reported ALRs (Johnston and Diven 1969; Inagaki et al. 1986; Kanda-Nambu et al. 2000; Ju et al. 2009; Kanodia et al. 2009; Liu et al. 2012). The amino acid sequences of *L. salivarius* UCC118, *B. pseudofirmus* OF4 and *G. stearothermophilus* IFO 12550 ALRs were retrieved from the UniProt data base (http://www.uniprot.org/), and that of *P. putida* YZ-26 was obtained from the report by Liu et al. (2012). The sequences of *B. anthracis* Sterne 34F2 (Kanodia et al. 2009), *B. subtilis* PCI219 (Kanda-Nambu et al. 2000) and *L. fermentum* ATCC9330 (Johnston and Diven. 1969) ALRs were not found in a data base, but those of *B*. *anthracis* ATCC14578, *B. subtilis* 168 and *L. fermentum* ATCC14931 were obtained from the UniProt data base and used as alternatives. Multiple alignments were performed using TCoffee (http://www.tcoffee.org/Projects/tcoffee/).

### Plasmid construction

The ORF containing the *ALR* gene in *L. salivarius* (Uniprot ID: Q1WV14) was amplified using PCR with chromosomal DNA from *L. salivarius* UCC118 and primers ALR-F 5′-ATATCATATGGTAATTGGAAGACATCG-3′ (*Nde*I site is underlined) and ALR-R 5′-ATTCTCGAGCTACTTGTAAATTCTTGGAAC-3′ (*Xho*I site is underlined). The PCR product was cloned between the *Nde*I and *Xho*I sites of pET-28a (Novagen, Darmstadt, Germany) to obtain pET-*ALR*.

To generate the *ALR*^*A131K*^ mutant gene, the upstream and downstream regions of *ALR* were amplified from pET-*ALR* using primers T7 promoter-F 5′-TAATACGACTCACTATAGGG-3′and ALRA131K-R 5′-CATACCTGTGTCTAATTTTAGGTGGATCTTTAATCTTTG-3′ (mutation bases are underlined) for the upstream, and primers ALRA131K-F 5′-CAAAGATTAAAGATCCACCTAAAATTAGACACACGTATG-3′ (mutation bases are underlined) and T7 terminator-R 5′-GCTAGTTATTGCTCAGCGG-3 for the downstream. The two fragments were subsequently fused using overlap extension PCR to give the mutant gene *ALR*^*A131K*^. The fused fragment was cloned between the *Nde*I and *Xho*I sites of pET-28a to give pET-*ALR*^*A131K*^.

### Preparation of recombinant proteins

*Escherichia coli* BL21-CodonPlusTM(DE3)-RIPL cell (Stratagene, CA, USA) harboring pET-*ALR* was cultured in 5 mL of LB medium containing 50 mg/L kanamycin and 50 mg/L chloramphenicol overnight at 37 °C. The cells were then subcultured and induced using 100 mL of Overnight Expression medium containing 45 g/L Overnight Express™ Instant LB Medium (Novagen), 50 mg/L kanamycin and 50 mg/L chloramphenicol for 18 h at 25 °C with shaking (180 rpm). The cells were then pelleted by centrifugation (8500×*g* for 10 min at 4 °C) and resuspended in 20 mM MOPS buffer (pH 7.0) containing 500 mM NaCl, after which the suspension was sonicated and again centrifuged as described above. The supernatant was then filtered (0.22 μm pore size), and the enzyme was purified on a Ni–NTA Agarose column (QIAGEN, Venlo, The Netherlands). The purified enzyme was dialyzed against 100 volumes of 20 mM MOPS buffer (pH 7.0) containing 500 mM NaCl and 2.0 mM EDTA for 12 h at 4 °C with two changes of the buffer solution. The resultant dialysate was then concentrated by ultrafiltration using an Amicon Ultra (Merck Millipore, MS, USA). The resultant enzyme solution was stored in the presence of 50 % glycerol at −20 °C. ALR^A131K^ protein was prepared using the same procedure with pET-*ALR*^*A131K*^.

### ALR assays

ALR and ALR^A131K^ activities were determined by measuring the initial velocity of d-alanine formation from l-alanine using ultra-performance liquid chromatography (UPLC) (Waters, Tokyo, Japan). The standard reaction mixture (0.1 mL) containing 100 mM MES buffer (pH 7.0), 20 mM l-alanine, 0.05 mM pyridoxal-5′-phosphate (PLP) and 10 % (v/v) purified enzyme solution. The enzyme reaction was run for 5 min at 30 °C and was then stopped by adding 0.1 mL of 20 % trichloroacetate. The UPLC analysis was performed with an ACQUITY UPLC TUV system consisting of a Waters Binary Solvent Manager, Sample Manager, FLR Detector and AccQ-Tag Ultra 2.1 × 100-mm column (Waters). The eluent flow rate was 0.20 mL/min, the column temperature was 45 °C, and the fluorescent wavelengths of the FLR detector were 350 and 450 nm. The eluent was linearly graduated using 80 % sodium acetate buffer (50 mM, pH 5.9) and 20 % methanol. All enzyme assays were performed more than three times under the same conditions. Mean values and standard deviations were calculated from each assay.

### Effect of temperature on activity

The effect of temperature on ALR and ALR^A131K^ activities was examined using the standard reaction mixture and varying the temperature between 20 and 60 °C in presence and absence of 1 mM acetate.

### Effect of pH on activity

The effect of pH on ALR and ALR^A131K^ activities was examined by measuring their activities under standard reaction conditions using different pH buffers in presence and absence of 1 mM acetate. The buffers used for the assays were 100 mM citrate (pH 4.0–5.5), 100 mM MES (pH 5.5–7.0), 100 mM Tris–HCl (pH 7.0–9.0) and 100 mM sodium carbonate (pH 9.0–11.0).

### Effect of various carboxylates on enzyme activity

The effects of various carboxylates and related carboxylates containing acetate, propionate, butyrate, d, l-lactate, citrate, d, l-glutamate, d, l-aspartate, pyruvate, 2-oxoglutarate, oxaloacetate succinate and fumarate on ALR and ALR^A131K^ activities were examined. All carboxylates used in this study were sodium salts or were neutralized with sodium hydroxide before use. The carboxylates (1 mM) were added to the standard reaction mixture, and the rate of l-alanine racemization was determined using the UPLC method as described above.

### Kinetic analysis

Steady state kinetic analyses of ALR and ALR^A131K^ were performed using various concentrations of l-alanine in the presence of several concentrations of acetate. Initial velocity was measured using UPLC to determine the rate of conversion of l-alanine to d-alanine. The l-alanine concentrations used were 6.67, 10.0, 13.3, 20.0 and 40.0 mM, and the acetate concentrations were 1.00, 10.0, 20.0 and 40.0 mM.

## Results

### Primary sequence and amino acid composition of ALRs from *L. salivarius* and other sources

**Fig. 1:**
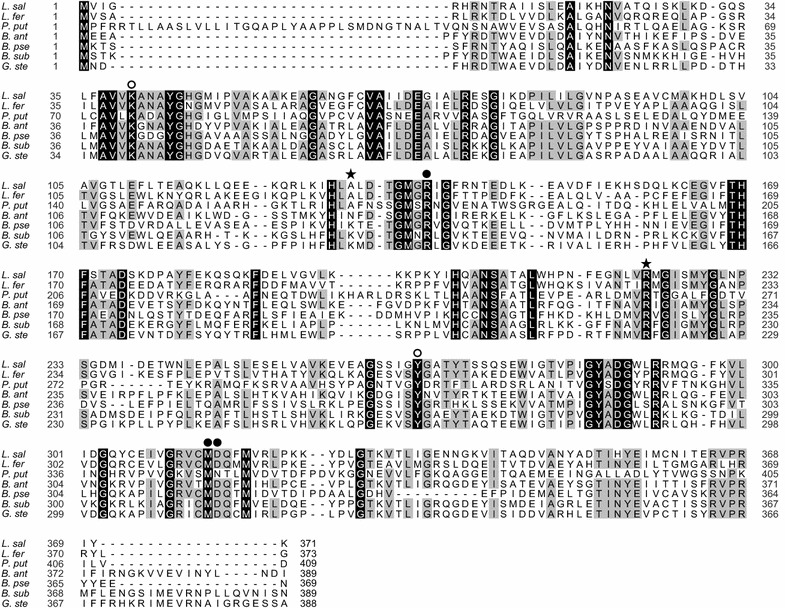
Amino acid sequence alignment of *L. salivarius* ALR with other ALRs: *L. sal*, *L. salivarius* UCC 118; *L. fer*, *L. fermentum* ATCC14931; *P. put*, *P. putida* YZ-26; *B. ant*, *B. anthracis* Sterne 43F2; *B. pse*, *B. pseudofirmus* OF4; *B. sub*, *B. subtilis* PCI 219; *G. ste*, *G. stearothermophilus* IFO 12550. The catalytic residues K40 and Y267, the carboxylate binding residues R138, M314 and D315, and A131 are marked with an *open circles*, *filled circles* and a *filled star*, respectively

We found a gene homologous (Q1WV14 in Uniprot) with ALR in an *L. salivarius* DNA database, and compared amino acid compositions of *L. salivarius* with other sources of ALRs (Table 1). The amino acid composition of the *L. salivarius* ALR was different from those of other ALRs in contents of arginine and lysine. However, affections to characteristics of *L. salivarius* ALR by these differences are unclear. Thus we aligned and compared the deduced amino acid sequence with those of ALRs from six other sources (Fig. 1). Although *L. salivarius* ALR showed relatively low overall sequence homology with ALRs from *B. anthracis* Sterne 43F2 (35.0 %), *B. pseudofirmus* OF4 (26.1 %), *B. subtilis* 168 (34.6 %), *G. stearothermophilus* IFO 12550 (35.6 %) and *P. putida* YZ-26 (20.4 %), the two catalytic bases in the active site, K40 and Y267, were strictly conserved (Tanizawa et al. 1988; Shaw et al. 1997; Watanabe et al. 2002). In addition, among the four residues (K129, R138, M314 and D315) reportedly responsible for the binding of carboxylates such as acetate and propionate, which are specific inhibitors in the case of ALRs from *Bacillus* and *Geobacillus* species (Morollo et al. 1999; Kanodia et al. 2009), R138, M314 and D315 are conserved in the sequence of *L. salivarius* ALR. On the other hand, K129 is replaced by A131 in *L. salivarius* ALR, which suggests carboxylates may exert a different effect on *L. salivarius* ALR.

**Table 1:** Amino acid composition of *L. salivarius* ALR with other ALRs

Amino acid	Number of residues per subunit						
	*L. sal*	*L. fer*	*P. put*	*B. ant*	*B. pse*	*B. sub*	*G. ste*
Alanine	33	41	51	32	36	37	39
Arginine	14	23	27	20	17	21	30
Asparagine	15	8	19	15	11	14	9
Aspartate	19	16	21	19	21	18	22
Cystein	6	4	2	1	5	5	4
Glutamate	14	15	14	8	9	8	10
Glutamine	27	27	22	30	27	28	24
Glycine	31	32	34	28	25	30	26
Histidine	10	7	11	8	13	11	16
Isoleucine	29	15	14	33	24	23	24
Leucine	30	36	42	31	35	38	39
Lysine	28	15	15	20	17	29	13
Methionine	11	12	11	6	11	14	10
Phenylalanine	12	13	13	21	13	14	16
Proline	11	16	12	17	14	15	23
Serine	20	15	25	16	29	19	17
Threonine	18	23	25	25	22	20	19
Tryptophan	4	3	4	6	0	4	5
Tyrosine	12	15	9	16	12	13	14
Valine	27	37	38	37	28	28	28

*L. sal* *L. salivarius* UCC 118, *L. fer* *L. fermentum* ATCC14931, *P. put* *P. putida* YZ-26, *B. ant* *B. anthracis* Sterne 43F2, *B. pse* *B. pseudofirmus* OF4, *B. sub* *B. subtilis* PCI 219, *G. ste* *G. stearothermophilus* IFO 12550

### Purification of *L. salivarius* ALR and ALR^A131K^ from recombinant *E. coli*

We cloned the *L. salivariusALR* gene (Q1WV14 in Uniprot) and its substitution mutant, *ALR*^*A131K*^, to obtain recombinant *E. coli* BL21-CodonPlusTM(DE3)-RIPL cells containing a hybrid plasmid harboring Q1WV14 or its *ALR*^*A131K*^ mutant gene. Both expression products were purified to homogeneity using one-step Ni–NTA agarose column chromatography with high yields of 60.7 and 51.6 %, respectively. Both his-tagged recombinant proteins were exhibited single band located with estimated molecular mass of 43 kDa (molecular mass of *L. salivarius* ALR from primary amino acid sequence is about 41 kDa). The activity of ALR^A131K^ was 438 ± 6.25 μmol/min mg, much higher than that of the ALR (161 ± 5.83 μmol/min mg) under the standard assay conditions.

### Effect of temperature on activity

**Fig. 2:**
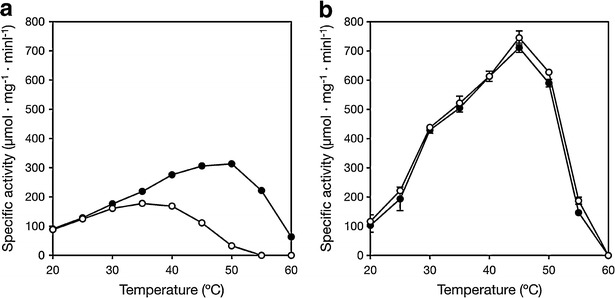
**a**, **b** Effect of temperature on ALR and ALR^A131K^ activities. ALR (**a**) and ALR^A131K^ (**b**) activities were assayed for 5 min at the indicated temperatures and pH 7.0 (MES buffer) using 20 mM l-alanine as the substrate with and without 1 mM acetate. For ALR, the maximum rates with and without acetate were 317 ± 5.72 and 178 ± 2.04 μmol/min mg, respectively (n = 3). For ALR^A131K^, the maximum rates with and without acetate were 711 ± 16.0 and 745 ± 23.4 μmol/min mg, respectively (n = 3). *Open circles*, without acetate; *filled circles*, with 1 mM acetate

The effects of temperatures between 20 and 60 °C on the activities of purified ALR and ALR^A131K^ were examined using 20 mM l-alanine as the substrate in the presence and absence of 1 mM acetate (Fig. 2a, b). The maximum activity of ALR without acetate was observed at 35 °C, but shifted to 50 °C in the presence of 1 mM acetate (Fig. 2a). Moreover, the activity of ALR in the presence of 1 mM acetate at 50 °C was much higher than in its absence at 35 °C (317 ± 5.72 μmol/min mg vs. 178 ± 2.04 μmol/min mg). In the absence of acetate, ALR activity was completely lost at 55 °C, but it exhibited about 71 % relative activity in the presence of 1 mM acetate. By contrast, 1 mM acetate had little or no effect on the temperature dependence of ALR^A131K^ activity, and maximum ALR^A131K^ activity was observed at 45 °C in presence or absence of acetate (Fig. 2b).

### Effect of pH on activity

**Fig. 3:**
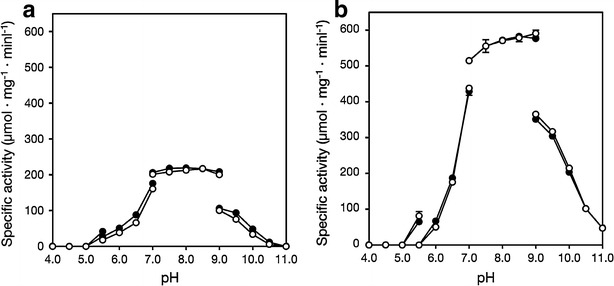
**a**, **b** Effect of pH on ALR and ALR^A131K^ activities. ALR (**a**) and ALR^A131K^ (**b**) activities were assayed for 5 min at 30 °C and the indicated pHs using 20 mM l-alanine as substrate. For ALR, the maximum rates with and without acetate were 219 ± 3.61 and 217 ± 3.25 μmol/min mg, respectively (n = 3). For ALR^A131K^, the maximum rates with and without acetate were 583 ± 0.593 and 591 ± 9.10 μmol/min mg, respectively (n = 3). *Open circles*, without acetate; *filled circles*, with 1 mM acetate. The buffers used were citrate (pH 4.0–5.5, MES (pH 5.5–7.0), Tris–HCl (pH 7.0–9.0) and carbonate (pH 9.0–11.0)

The effect of pH on ALR and ALR^A131K^ activities was examined at pH 4.0–11.0 in the presence and absence of 1 mM acetate (Fig. 3a, b). ALR showed maximum activity at around pH 8.0 in presence and absence of 1 mM acetate. In addition, the relative activity of ALR was slightly increased by the addition of acetate. On the other hand, ALR^A131K^ exhibited maximum activity at around pH 9.0, and ALR^A131K^ activity was unaffected by 1 mM acetate at any pH.

### Kinetic analyses of *L. salivarius* ALR

**Fig. 4:**
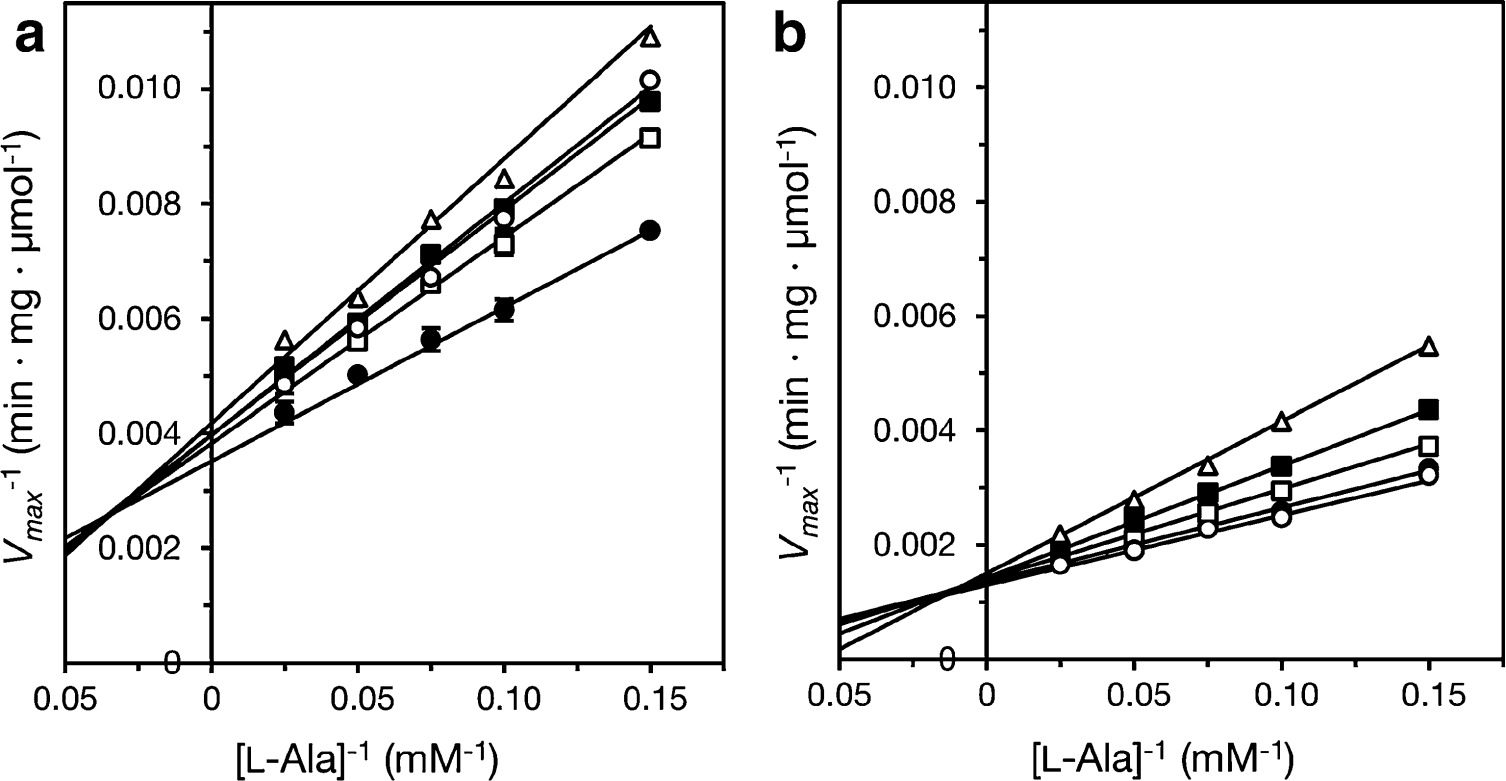
Kinetics of acetate inhibition of ALR and ALR^A131K^. **a**, **b** ALR and ALR^A131K^ were assayed for 5 min at 30 °C and pH 7.0 (MES buffer) using various concentrations of l-alanine and acetate as the substrate and inhibitor, respectively. Shown are double reciprocal plots of the initial velocity of ALR (**a**) or ALR^A131K^ (**b**) against l-alanine concentrations at several concentrations of acetate (n = 3 for each enzyme). *Open circles*, without acetate; *filled circles*, 1.0 mM acetate; *open squares*, 10.0 mM acetate; *filled squares*, 20.0 mM acetate; *open triangles*, 40.0 mM acetate

The initial velocity of d-alanine formation from l-alanine catalyzed by ALR was measured at several l-alanine concentrations in the presence of five concentrations of acetate. The resultant Lineweaver–Burk plots of the relation between the l-alanine concentration and the initial velocity showed five straight lines (Fig. 4a, b). Although ALR was strongly activated by the addition of 1 mM acetate, the activation level was reduced by the addition of acetate concentrations above 10, and 40 mM acetate greatly inhibited ALR activity at all l-alanine concentrations (Fig. 4a). By contrast, ALR^A131K^ activity was inhibited or unaffected at all the acetate contractions tested (1–40 mM). The *K*_m_ values of ALR and ALR^A131K^ for l-alanine in absence of acetate were 11.5 and 9.20 mM, respectively. The *V*_max_ of ALR and ALR^A131K^ in absence of acetate were 272 and 751 μmol/min mg, respectively.

### Effect of different carboxylates on the activity

**Fig. 5:**
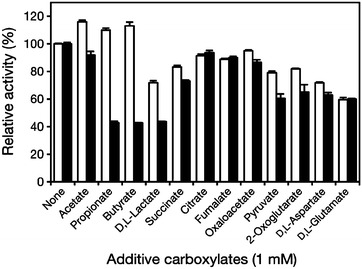
Effect of various carboxylates on ALR and ALR^A131K^ activities. ALR and ALR^A131K^ were assayed for 5 min at 30 °C and pH 7.0 (MES buffer) using 20 mM l-alanine as the substrate in the presence of the indicated carboxylates (1 mM). The specific activities of ALR and ALR^A131K^ at 100 % were 171 ± 0.559 and 527 ± 5.17 μmol/min mg, respectively (n = 3). *Open bars*, ALR activities; *filled bars*, ALR^A131K^ activities

We also tested the effect of carboxylates other than acetate on the activities of ALR and ALR^A131K^ (Fig. 5). ALR^A131K^ was inhibited to a greater degree than ALR by several of the carboxylates tested. Although propionate and butyrate are reportedly strong inhibitors of *Bacillus* and *Geobacillus* ALRs (Morollo et al. 1999; Kanodia et al. 2009), they strongly activated *L. salivarius* ALR. By contrast, both propionate and butyrate strongly inhibited ALR^A131K^. ALR and ALR^A131K^ were both inhibited by d, l-lactate, though ALR was inhibited to a lesser degree than ALR^A131K^. In addition, succinate, pyruvate, 2-oxoglutarate, oxaloacetate and aspartate also more strongly inhibited ALR^A131K^ than ALR.

## Discussion

To better understand the mechanism responsible for the high yield of d-alanine in *L. salivarius*, we characterized the ALR expressed by *L. salivarius*, which may be entirely responsible for its d-alanine production. We found that the fundamental properties of this enzyme, including its temperature and pH optima and kinetic parameters, are similar to those of ALRs from other sources such as *B. pseudofirmus* and *P. putida* (Ju et al. 2009; Liu et al. 2012). Nonetheless, the sequence homologies between *L. salivarius* ALR and the ALRs from other sources were not high, and the *L. salivarius* enzyme was activated by several short-chain carboxylates (acetate, propionate and butyrate) that inhibit other ALRs. In particular, this activation is in contrast *G.stearothermophilus* ALR, which is clearly inhibited by both acetate and propionate (Morollo et al. 1999).

Six amino acid residues are responsible for the activity of *G.stearothermophilus* ALR (Morollo et al. 1999). Among the corresponding residues in *L. salivarius* ALR, five are conserved, but K129 of *G.stearothermophilus* ALR is replaced by A131 in *L. salivarius* (Fig. 1). We therefore prepared and characterized an A131K variant of *L. salivarius* ALR. Interestingly the ALR^A131K^ variant enzyme exhibited much greater activity than the wild-type enzyme; moreover, the activation by acetate and propionate disappeared, replaced by a typical mix-type inhibition same as previous reports of *Bacillus* ALR (Kanodia et al. 2009). This suggests acetate and propionate may be involved in the control of ALR activity, and that A131 may be responsible for the regulation of d-alanine synthesis in *L. salivarius*.

In *G. stearothermophilus* ALR, K129 reportedly interacts with R136, which binds to carboxylate and PLP oxygen through formation of carbamate (Morollo et al. 1999). This interaction between the K129 and R136 residues through carbamylation has also been seen in *P. aeruginosa* ALR (LeMagueres et al. 2003), and the same interaction occurs in *Staphylococcus aureus* ALR, though a sulfate ion substitutes for the carbamylation (Scaletti et al. 2012). Similarly, an equivalent N129 residue of *B. anthracis* ALR interacts with R136 through a chloride ion (Couñago et al. 2009). From these reports, it appears that there is often an interaction between K129 or N129 and R136 mediated in various ways within ALR. However the alanine side chain has no charge, so there is no interaction between A131 and R138 in *L. salivarius* ALR (which corresponds to R136 in *Bacillus* ALR). Consequently, R138 of *L. salivarius* ALR is probably poorly ordered and what would be the carboxyl group binding site is unstable. Acetate may stabilize *L. salivarius* ALR by binding to the carboxyl group binding site, and the activating effect of this structural stabilization may be more pronounced than acetate’s inhibitory effect so that the net effect on *L. salivarius* ALR activity is stimulation. Consistent with that idea is the dramatic increase in ALR activity and stability seen in the A131K variant (Fig. 2), as well as the activating and inhibitory effects of acetate on ALR activity (Fig. 4). These stabilizing effect of competitive inhibitors were found in other enzymes (Burton 1951; Alvaro et al. 1991). A more precise explanation of the structural basis of the activation and/or stabilization of *L. salivarius* ALR will require analysis of its crystal structure.

Zalan et al. reported the robust production of organic acids such as lactate and acetate by various *Lactobacillus* species (Zalán et al. 2010). In addition, propionate and butyrate are also produced by some *Lactobacillus* strains (Liong and Shah 2005; Pereira et al. 2003). Notably, when milk and Jerusalem artichoke juice are used for the growth medium, lactate production is depressed in many *Lactobacillus* strains, whereas acetate production is enhanced. As a result, the acetate concentration in cells from some *Lactobacillus* strains is higher than the lactate concentration, particularly under aerobic culture conditions (Bobillo and Marshall 1991; Cselovszky et al. 1992). This suggests that carboxylates such as acetate, propionate and butyrate might play regulatory roles affecting d-alanine production in *Lactobacillus* strains, through details of the control of ALR activity remain to be determined.

## Abbreviations

ALR: alanine racemase
ALR^A131K^: alanine racemase A131K variant
PLP: pyridoxal 5′-phosphate
UPLC: ultra-performance liquid chromatography
